# Intraoperative magnesium sulphate as an analgesic adjunct in a multimodal, opioid-sparing protocol for canine ovariohysterectomy: a prospective, randomized, controlled, double-blind study

**DOI:** 10.3389/fvets.2026.1771080

**Published:** 2026-05-13

**Authors:** Stefanos G. Georgiou, Filimon Diamantis, Tilemachos L. Anagnostou, Fotini A. Koutina, Evgenia Papagiorgou, Theodora Κ. Milini, Apostolos D. Galatos

**Affiliations:** 1 Clinic of Surgery, Faculty of Veterinary Medicine, University of Thessaly, Karditsa, Greece; 2 Volos Veterinary Specialists, Volos, Greece; 3 Companion Animal Clinic, Faculty of Veterinary Medicine, Aristotle University of Thessaloniki, Thessaloniki, Greece

**Keywords:** analgesia, dog, magnesium sulphate, multimodal analgesia, ovariohysterectomy, postoperative pain

## Abstract

**Introduction:**

Magnesium acts as a non-competitive NMDA receptor antagonist and may attenuate central sensitization. Evidence regarding its analgesic- or anaesthetic-sparing effects in dogs remains inconclusive. This prospective, randomized, blinded, controlled clinical study evaluated the effect of intraoperative magnesium sulphate on postoperative pain in dogs undergoing ovariohysterectomy (OVH).

**Methods:**

Twenty-eight bitches were randomly allocated to receive magnesium sulphate (*n* = 14; 50 mg kg^−1^ IV dose followed by 20 mg kg^−1^ h^−1^) or saline (*n* = 14). An *a priori* power analysis (*α* = 0.05, power = 0.8) determined that 28 dogs were required to detect a 2-point difference in the short-form Glasgow Composite Measure Pain Scale (CMPS-SF) scores (SD 1.8; Cohen’s *d* = 1.11). Anaesthesia was induced with propofol and maintained with isoflurane. Postoperative pain (primary outcome) was assessed using the CMPS-SF for 48 h. Continuous variables were analyzed using independent-samples *t*-tests or repeated-measures ANOVA with Bonferroni correction; categorical variables were compared using Chi-square or Fisher’s exact test. Significance was set at *p* < 0.05.

**Results:**

All dogs completed the study (*n* = 28). Mean CMPS-SF scores at 1 h post-extubation were significantly lower in the Magnesium group compared with the Control group (3.9 ± 1.5 vs. 5.8 ± 1.9; *p* = 0.012). Pain scores varied over time (*F* = 72.368, *p* < 0.01), with a significant time x treatment interaction (*F* = 2.851, *p* = 0.004). Intraoperative fentanyl requirements (0.3 ± 0.6 vs. 0.6 ± 0.8 μg kg^−1^; *p* = 0.275) and postoperative rescue analgesia rates (21.4% vs. 35.7%; *p* = 0.673) did not differ between groups. The propofol induction dose was significantly lower in the Magnesium group (2.8 ± 0.5 vs. 3.7 ± 0.4 mg kg^−1^; *p* < 0.01), whereas end-tidal isoflurane concentrations were similar (*p* = 0.112). Extubation time and time to first head movement were prolonged in the Magnesium group (*p* < 0.01). Postoperative serum magnesium concentrations were significantly higher in the Magnesium group (*p* < 0.01), while adverse effects did not differ between groups (35.7% vs. 21.4%; *p* = 0.683).

**Conclusion:**

Intraoperative magnesium administration in dogs undergoing OVH, within a multimodal protocol, improved early postoperative analgesia and reduced propofol requirements without an opioid- or isoflurane-sparing effect. The dosing regimen was well tolerated, although recovery duration was prolonged. Clinical impact may be more evident in more invasive procedures or opioid-free approaches.

## 1 Introduction

Magnesium is the second most abundant intracellular cation after potassium and the fourth most prevalent element in the body. Over 99% is located intracellularly, primarily within skeletal muscle and bone, while < 2% is present in the systemic circulation in ionized (biologically active), protein-bound, or anion-complexed forms (1–3). The ileum is the principal site of magnesium absorption, with additional uptake occurring in the jejunum and colon. Magnesium homeostasis is maintained by the kidneys, which regulate its reabsorption or excretion to ensure appropriate physiological balance (1, 2, 4, 5). Magnesium is essential for numerous biological processes, including enzymatic reactions involved in metabolism and protein synthesis, as well as for maintaining cellular integrity and function, playing a key role in apoptosis prevention (1–3). Disturbances in magnesium homeostasis have also been linked to increased morbidity and mortality in critically ill human and veterinary patients (1). Accurate assessment of total body magnesium is challenging due to its predominantly intracellular location, and measurement relies on either total or ionized magnesium concentrations, with ionized magnesium preferred; low ionized magnesium levels generally seem to reflect low total magnesium (1).

Magnesium is tightly regulated in the body, and perioperative disturbances in magnesium homeostasis may occur due to factors such as preoperative fasting, surgical stress, or perioperative administration of magnesium-free fluids (6). Hypomagnesaemia has been associated with complications in various species, including prolonged hospitalization in dogs and cats or postoperative ileus in horses (1, 7, 8). Conversely, hypermagnesaemia most commonly arises from renal impairment, urinary obstruction or iatrogenic administration (1, 4, 8). Elevated serum magnesium may enhance neuromuscular blockade, by inhibiting presynaptic calcium channels and acetylcholine release, leading to flaccid muscle paralysis, reduced or absent spinal reflexes, respiratory depression, mild to moderate hypotension, bradycardia, flushing, gastrointestinal symptoms, coma and cardiac arrest (3, 5, 9). Dogs with hypermagnesaemia have a 2.6-fold higher mortality risk, and neuromuscular effects can occur even with mild magnesium elevations (4, 7). In case of iatrogenic hypermagnesaemia, serum magnesium levels typically normalize rapidly once administration ceases, provided renal function is intact (4).

Therapeutic magnesium administration has been investigated in critically ill patients for the management of reperfusion syndrome, systemic inflammatory response (SIRS), bronchospasm, tetanus, traumatic brain injury, preeclampsia, and as an adjunct in general and regional anaesthesia (1, 3, 9). In these settings, magnesium administration is not aimed at correcting deficiency, but at exploiting its beneficial effects on specific cell types (1). Magnesium is generally safe with a wide therapeutic window; serious toxicity usually occurs only when serum concentrations exceed five times the normal range (3). Magnesium is now recognized as essential, with deficiency adversely affecting numerous physiological functions, and perioperative hypomagnesaemia should be avoided, particularly in critically ill patients (9).

Magnesium acts as both a physiological and pharmacological inhibitor of N-Methyl-D-Aspartate (NMDA) receptors, which play a key role in pain modulation, and is increasingly used in the management of both acute and chronic pain states (9). Its analgesic mechanism is primarily through NMDA-receptor antagonism, targeting pathways involved in pain transmission. NMDA receptors are voltage-gated ion channels in the central nervous system that allow sodium and calcium influx and potassium efflux. Under resting conditions, magnesium blocks these receptors, preventing calcium entry, whereas the release of glutamate and other neurotransmitters can depolarize the neuronal membrane, allowing channel opening. Activation of NMDA receptors and the resulting rise in intracellular calcium contribute to central sensitization and the “wind-up” phenomenon, both of which play key roles in chronic pain mechanisms. Magnesium, by antagonizing NMDA receptors, therefore, appears to prevent the establishment of central sensitization and further potentiation of pain (2, 10, 11). Clinical studies in humans report beneficial effects of intravenous (IV) magnesium in conditions such as migraine, abdominal pain, peripheral neuropathy and complex regional pain syndrome (CRPS) (2, 11). However, because magnesium does not appear to cross the human blood–brain barrier, the precise way of its therapeutic action, central, spinal or peripheral, remains unclear (12). Moreover, magnesium does not seem to exert primary analgesic effects; instead, it appears valuable as an adjunctive agent enhancing the efficacy of other analgesics (9).

In human literature, magnesium is administered IV at a loading dose of 30–50 mg kg^−1^, followed by continuous infusion of 6–20 mg kg^−1^ h^−1^ until the end of the surgical procedure or for up to 4 h after the initial bolus, when aiming perioperative analgesic management. Absorption after IV administration appears to be rapid, and any analgesic effect is reported to be immediate, with an approximate duration of 30 min (2). Perioperative IV magnesium reduces postoperative pain levels and opioid consumption, whether administered as a single bolus or followed by continuous infusion (2, 11, 13), with current evidence not indicating superiority of one administration method over the other (12, 14, 15).

In humans, the first randomized controlled study (RCT) reporting a beneficial effect of magnesium (16), in which magnesium was administered as an IV loading bolus followed by continuous infusion, demonstrated that patients receiving magnesium required approximately 30% less morphine in the first 48 postoperative hours, with the greatest reduction occurring in the first 6 h. Systematic reviews and meta-analyses confirm that adjunctive magnesium reduces postoperative pain and opioid use in the first 24 h after surgery (14, 15). A meta-analysis of 1,461 patients reported a 24.4% reduction in morphine consumption with perioperative IV magnesium, independent of administration method and without major adverse effects (14). De Oliveira et al. (15) similarly found reduced morphine requirements and lower pain scores, supporting magnesium as an effective adjunct. A later meta-analysis showed a modest but significant reduction in postoperative morphine use (approximately 5 mg) in non-cardiac surgery (6). Across studies, magnesium was administered using variable regimens, including single bolus doses, continuous infusions, or a combination of both. Retrospective data also indicate benefits within opioid-free anaesthesia, with lower postoperative pain, reduced rescue analgesia and less nausea and vomiting when magnesium and ketamine were used instead of remifentanil (10). However, a 2021 RCT found no postoperative analgesic advantage over remifentanil, with more magnesium-treated patients requiring intraoperative rescue opioids, and similar postoperative pain scores between groups (13). Regarding magnesium-related adverse effects, although bradycardia was reported as the most common symptom, it was transient and responded to standard treatment with no reports of persistent haemodynamic instability (14). In the study of Tramer et al. (16), patients with normal renal function exhibited rapid magnesium clearance, and haemodynamics were similar to controls intraoperatively and postoperatively. A meta-analysis by Ng et al. (6), reported significantly higher magnesium levels in treated patients, reduced shivering and no significant differences in postoperative nausea, vomiting, or bradycardia. Similarly, a recent RCT found that only 13% of magnesium-treated patients required intervention for hypotension versus 60% in the remifentanil group, suggesting improved intraoperative haemodynamic stability with magnesium during TIVA (13). Minimizing the duration of IV magnesium administration has been recommended by the authors to prevent excessively high magnesium concentrations during the perioperative period (13).

Veterinary literature includes clinical trials in dogs (17–27) that investigate the effect of magnesium administration during the perioperative period; however, the results remain inconclusive. Additionally, a recent qualitative review evaluating the analgesic- and anaesthetic-sparing effects of magnesium concluded that evidence supporting its perioperative adjunctive role within a multimodal approach is weak, owing to the limited amount and low quality of the available data (28).

In dogs, magnesium has been administered IV in both RCTs (17–19, 22, 25–27) and case reports (29, 30), as well as via epidural or spinal routes (20, 21, 23) and intraperitoneally (24). For IV administration, loading doses of 40–50 mg kg^−1^ followed by continuous infusions of 10–80 mg kg^−1^ h^−1^ have been described (17–19, 22, 25–27, 29, 30). All published studies using IV magnesium have focused on acute perioperative nociceptive pain following surgical procedures. These include OVH (17, 19, 27), enterotomy (18), tibial plateau leveling osteotomy (25), orchiectomy (26), and phaeochromocytoma resection (29, 30). Among the RCTs employing IV magnesium, some did not evaluate its analgesic effects (18, 22, 26) and some were not conducted under clinical conditions involving a nociceptive stimulus (22, 26). Concerning the two case reports, magnesium sulphate was administered at 40–50 mg kg^−1^ followed by continuous infusion of 15 mg kg^−1^ h^−1^ within a multimodal approach to manage nociception and haemodynamic disturbances associated with acute catecholamine release during phaeochromocytoma resection (29, 30).

However, there are no established dosing recommendations for IV magnesium administration as an analgesic agent in dogs, and the regimens used to date have largely been extrapolated from human medicine. Studies evaluating the perioperative analgesic effects of IV magnesium have assessed its impact on intraoperative (17, 25, 27) or postoperative rescue analgesia requirements (17, 19, 25, 27), or postoperative pain scores (17, 19, 25). In two RCTs involving 46 and 16 dogs undergoing OVH, IV magnesium did not contribute to a clinical advantage (17, 19). Anagnostou et al. (17) reported no differences in intraoperative or postoperative rescue analgesia requirements between dogs receiving magnesium sulphate (50 mg kg^−1^ as a loading bolus, followed by a 12 mg kg^−1^ h^−1^ infusion) and the control group. Similarly, Rioja et al. (19) found no evidence that intraoperative IV magnesium (50 mg kg^−1^ followed by 15 mg kg^−1^ h^−1^) improved postoperative analgesia, as postoperative pain scores were similar between groups. In contrast, a more recent study found that dogs undergoing OVH and receiving magnesium sulphate (a 50 mg kg^−1^ loading bolus followed by an 80 mg kg^−1^ h^−1^ intraoperative infusion) demonstrated reduced intraoperative opioid consumption compared to the control saline solution group (27). Similarly, another recent study demonstrated a synergistic effect of magnesium and ketamine when co-administered as a continuous IV infusion during surgery. Dogs receiving the magnesium-ketamine combination required less intraoperative rescue analgesia and exhibited lower postoperative pain scores than those receiving ketamine alone during tibial plateau leveling osteotomy (25).

Regarding the effect of IV magnesium on intraoperative anaesthetic requirements, findings remain inconsistent, to some extent. Some studies have reported propofol- (18, 26), thiopental- (17) and halothane-sparing effects (17) following IV magnesium administration, whereas others have found no effect on propofol (27), isoflurane (19) or sevoflurane (22) requirements. The studies demonstrating anaesthetic-sparing effects employed a loading dose of 50 mg kg^−1^ IV, followed by continuous infusions ranging from 10 to 80 mg kgh^−1^. Conversely, Boff et al. (27) found no propofol-sparing effect of a continuous infusion of 80 mg kg^−1^ h^−1^; Rioja et al. (19) reported no isoflurane-sparing effect using a 50 mg kg^−1^ bolus followed by a 15 mg kg^−1^ h^−1^ infusion, and Johnson et al. (22) also found no reduction in sevoflurane requirements after a 45 mg kg^−1^ loading dose followed by a 15 mg kg^−1^ h^−1^ infusion. Although occasional magnesium-related adverse effects have been described, including vomiting, hypersalivation, nausea, or prolonged duration of anaesthesia (17, 18), most studies, including the latter two, reported no clinically significant cardiovascular or respiratory complications (17–19, 26), smooth and uneventful recoveries (18, 19, 22), or even faster extubation times (26).

The study aimed to evaluate the effect of intraoperative IV magnesium sulphate administration (50 mg kg^−1^ IV loading dose followed by a continuous infusion of 20 mg kg^−1^ h^−1^) as an adjunctive analgesic on postoperative pain intensity in dogs undergoing OVH, assessed using the short-form Glasgow Composite Measure Pain Scale (CMPS-SF) (31). Secondary objectives were to investigate its effects on intraoperative and postoperative analgesic requirements, anaesthetic requirements, intraoperative physiological responses, the incidence of perioperative adverse effects, serum magnesium concentrations, neuromuscular function (patellar reflex) and recovery quality. The study’s initial hypothesis was that incorporating a non-opioid agent such as magnesium sulphate into a multimodal, opioid-sparing anaesthetic approach would reduce postoperative pain intensity compared with control treatment, and decrease perioperative opioid requirements, lowering postoperative pain scores without increasing the incidence of adverse effects.

## 2 Materials and methods

### 2.1 Study design

**Figure 1:**
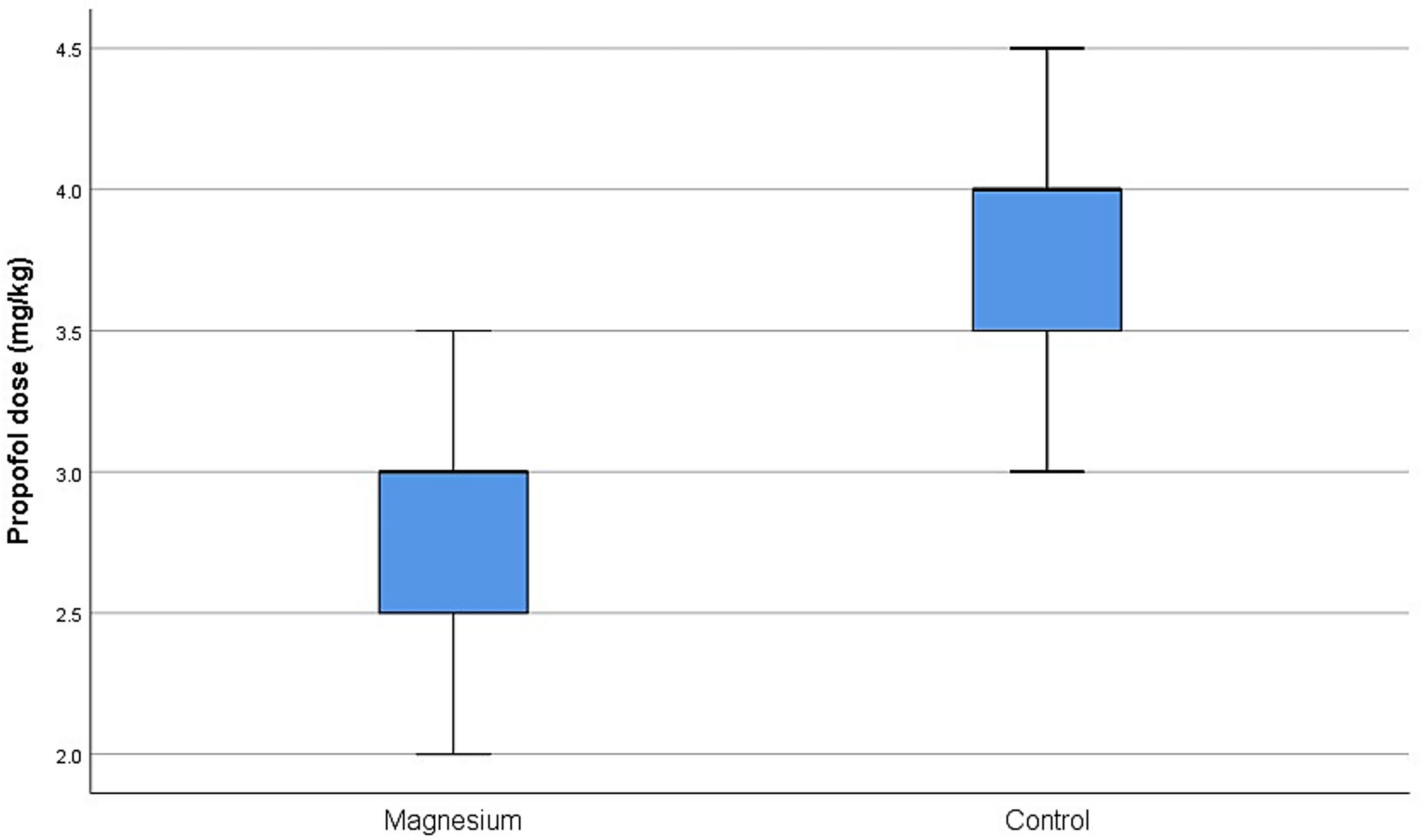
Boxplot presenting the propofol dose required for tracheal intubation in dogs administered magnesium sulphate or saline during the intraoperative period (*p* < 0.01).

This was a prospective, randomized, double-blind, controlled clinical study which was designed to investigate the effect of IV magnesium sulphate administration (an initial bolus followed by continuous infusion) on postoperative pain in client-owned dogs undergoing elective OVH. The study was carried out at the Volos Veterinary Specialists facilities, in Volos, and all procedures were performed in accordance with national veterinary practice standards and Greek animal welfare legislation (Law 4039/2012, as amended by Law 4235/2014). The study protocol was reviewed and approved by the Animal Ethics Committee of the Faculty of Veterinary Science, University of Thessaly (license number 142/12.07.2022), and the authors complied with the CONSORT guidelines for randomized controlled trials (32). A flow diagram of the study design is presented in Figure 1. A written informed owner’s consent was obtained before each dog’s inclusion to the study.

### 2.2 Animals

The study involved a total of 28 female, client-owned dogs of various breeds that were scheduled to undergo elective OVH. Clinically healthy dogs (ASA physical status 1), aged ≥ 6 months and < 8 years, weighing > 5 kg and < 30 kg, and appropriately vaccinated and dewormed were included. Dogs with unremarkable findings on physical examination and preoperative haematological and biochemical screening, including serum magnesium levels, were also eligible for enrollment. Only cooperative dogs that did not display excessive stress, fear or aggression during interactions with the clinical personnel were enrolled in the study. Exclusion criteria included a history of prior abdominal surgery, obesity or cachexia (body condition score > 4/5 or < 3/5), pregnancy, lactation, the pre-existence of acute or chronic pain conditions, history of neurological disorders (i.e., seizures) as well as the administration of sedatives, NSAIDs, opioids, corticosteroids or NMDA antagonists within the preceding 60 days.

### 2.3 Randomization and blinding

Dogs meeting the inclusion criteria, not falling under any exclusion criteria, and whose owners provided written informed consent were randomly assigned, using an online randomization tool (research randomizer, http://www.randomizer.org), to either the Magnesium group, in which magnesium sulphate was administered IV during the intraoperative period as an adjunct to the standardized anaesthetic protocol, or to the Control group which received the same standardized protocol plus IV saline. If a dog was excluded during the study, the next enrolled animal was assigned to the same treatment. Computerized randomization revealed the allocated intervention to a designated member of the research team, who was the only individual aware of group allocation. This person was responsible for preparing the intraoperative intervention (either the appropriate volume of magnesium sulphate or the equivalent volume of saline) but was not otherwise involved in the study procedures. Both the outcome assessor and the personnel involved in perioperative care (the anaesthetist and the surgeon) were blinded to treatment allocation. The dog owners were also unaware of the type of treatment their pet received, preserving the double-blind nature of the study. Additionally, the statistician responsible for data analysis was blinded to the group assignments and the overall study design.

### 2.4 Study protocol

Magnesium sulphate (Magnesium Sulfate 25%, Cooper, Greece) was administered to dogs in the magnesium group at a dose of 50 mg kg^−1^ as a slow IV bolus over a minimum of 15 min, immediately before propofol induction. The bolus was delivered via syringe pump (YSZS-SP01, Ysenmed, China) through a cephalic vein catheter into a running IV fluid line [lactated ringer’s solution at 5 mL.kg.h^−1^ (Lactated Ringer’s Solution, Vioser S.A, Greece)], allowing continuous dilution during administration. This was followed by a continuous IV infusion of 20 mg kg^−1^ h^−1^, maintained until the end of surgery. The maintenance infusion rate was selected based on previously published veterinary and human perioperative studies evaluating magnesium as an adjunct analgesic, representing the upper range of clinically reported regimens. In the control group, an equivalent volume of 0.9% NaCl (Sodium Chloride 0.9%, Vioser S.A, Greece) was administered following the same timing and infusion protocol. Both the magnesium sulphate and saline infusions were delivered via a syringe pump. The total volume to be infused (magnesium or saline) was pre-prepared each time by the same designated individual, ensuring that the anaesthesiologist remained blinded to the type of solution, as previously described.

### 2.5 Procedure (preoperative period, anaesthesia and surgery)

Dogs were admitted to the clinic approximately 2 h before surgery to allow for acclimatization to the clinical environment and personnel, as well as for preoperative evaluation and blood sampling. Following clinical assessment and sample collection, the dogs remained in a quiet environment until the administration of premedication. An 8-h preoperative fasting and a 2-h water restriction were implemented prior to surgery. Dogs were premedicated with dexmedetomidine (Dexdomitor, Orion Pharma, Finland) at 4 μg kg^−1^ and buprenorphine (Bupaq, Neocell, Greece) at 20 μg kg^−1^, administered IM. Twenty minutes later, the cephalic vein was aseptically catheterized and Lactated Ringer’s solution was initiated at a rate of 5 mL.kg.h^−1^. A single injection of cefuroxime (Zinacef, GlaxoSmithKline, Greece) at 20 mg kg^−1^ and meloxicam (Metacam, Boehringer Ingelheim, Germany) at 0.1 mg kg^−1^ were administered IV. Magnesium sulphate or the equivalent volume of saline was administered at that time, over at least 15 min, before induction of anaesthesia. Oxygen was delivered at 4 L.min^−1^ via facemask for 5 min before and during induction. Anaesthesia was induced with IV propofol (Propofol, Fresenius Kabi, Greece) increments of 1 mg kg^−1^ up to 2 mg kg^−1^, followed by 0.5 mg kg^−1^ every 30 s until fulfillment of the predefined criteria of tracheal intubation (loss of jaw tone, absence of resistance to the tongue protraction and absence of gag reflex and successful trachea intubation) with a proper diameter tube. The previously mentioned criteria for tracheal intubation were assessed after each propofol bolus and the total propofol dose required for intubation was recorded. Following intubation, the endotracheal tube was connected to a circle anaesthetic circuit, and anaesthesia was maintained with a mixture of isoflurane (Isoflo, Abbott, UK) in oxygen 100%. Afterwards, the dog was positioned in dorsal recumbency and prepared for surgery. Haemoglobin oxygen saturation (SpO_2_), heart rate (HR), respiratory rate (RR), noninvasive arterial (systolic, mean, diastolic) blood pressure (BP) measured by oscillometry using an appropriately sized cuff placed on the antebrachium, partial pressure of expired carbon dioxide (PETCO_2_), inspired and expired isoflurane concentrations, and rectal temperature were constantly monitored and recorded every 5 min intraoperatively until the end of anaesthesia, using a multiparametric monitor (Datex Ohmeda S/5, Datex-Ohmeda Division, Instrumentarium Corp, Finland). The temperature was strictly maintained between 37 and 38 °C by means of use of a forced-air heating system, whenever needed. Spontaneous ventilation was permitted during anaesthesia, except for the case that PETCO_2_ > 50 mmHg, when mechanical ventilation was initiated to maintain normocapnia, with that intervention being recorded. A routine midline OVH was performed by the same experienced veterinary surgeon each time who was blinded to the infused solution, and surgery was initiated only after each dog had achieved the desired anaesthetic depth (absence of palpebral reflex and jaw tone). The isoflurane vaporizer dial was adjusted to achieve a target end-tidal isoflurane concentration of 1.3%, maintained for at least 5–10 min before surgery initiation, which corresponds to MAC of isoflurane as has been determined in dogs (33). During surgery the desired anaesthetic depth was assessed based on neurological evaluation (eye position, palpebral reflex, corneal reflex, loss of jaw tone), while acute autonomic responses (acute or abrupt increases in HR, RR, BP) associated with surgical stimulation were used to monitor the analgesic adequacy. If clinical assessment of anaesthetic depth based on reflexes and jaw tone indicated a decrease of anaesthetic depth, the isoflurane concentration (dial setting) was increased by 0.2%. In case of dramatic anaesthetic depth reduction, propofol 1 mg kg^−1^ IV was administered and the dog was excluded from the study due to unacceptable anaesthetic depth fluctuation compromising study uniformity. Any abrupt increase in physiologic parameters (HR, RR, mean BP) > 20% was considered indicative of inadequate analgesia, and fentanyl (Fentanyl, Janssen–Cilag, Greece) 1 μg kg^−1^ IV was administered as rescue analgesia. In such cases, the surgical stimulus was ceased and not re-applied until the altered parameter returned to baseline. After a 5-min delay, parameters were re-evaluated, and the procedure continued only if they normalized; otherwise, another fentanyl bolus of 1 μg kg^−1^ was administered, with an additional 5-min delay. If analgesia remained inadequate, a continuous fentanyl infusion of 5–10 μg kg.h^−1^ was initiated, and the dog was excluded from the study due to protocol failure and unacceptable prolongation of surgical duration. Intraoperative data were recorded at predefined timepoints; T_0_ (baseline, 5–10 min at 1.3% FE_ISO_, just before application of the nociceptive stimulus), T_1_ (after first skin incision), T_2_ and T_3_ (after ligation of the 1st and 2nd ovarian pedicles, respectively), T_4_ (during abdominal wall closure), and T_5_ (after final skin suture). Bradycardia and hypotension were defined as HR < 60 beats/min or mean BP < 60 mmHg for more than 5 min and were treated with atropine 0.02 mg kg^−1^ IV or dopamine 7 μg kg.min^−1^, respectively. This threshold was selected in accordance with contemporary veterinary anaesthesia monitoring guidelines and specialist consensus identifying mean BP values around 60–65 mmHg as clinically relevant thresholds for intraoperative intervention (34, 35). At the end of surgery, paracetamol (Apotel plus, Uni-Pharma, Greece) 10 mg kg^−1^ IM was administered to all dogs. Postoperative analgesia consisted of oral paracetamol (Apotel, Uni-Pharma, Greece) 10 mg kg^−1^ administered twice daily and meloxicam 0.1 mg kg^−1^ once-a-day for 5 days. Dogs recovered in a warm and quiet room under continuous monitoring. In cases of rough or dysphoric recovery, propofol 0.5 mg kg^−1^ IV was administered; if inadequate, an additional 0.5 mg kg^−1^ IV propofol bolus was re-administered along with buprenorphine 10 μg kg^−1^ IV, as rescue analgesia. Dogs were discharged 48 h after extubation.

### 2.6 Outcome measures

The primary outcome of this study was postoperative pain intensity, assessed using the CMPS-SF at predetermined postoperative timepoints, until 48 h after emergence from anaesthesia.

Secondary outcomes included intraoperative and postoperative analgesic efficacy, intraoperative physiological effects, incidence of adverse effects during the perioperative period and recovery quality. Specifically, secondary outcomes comprised the number of dogs requiring rescue analgesia during the intraoperative and postoperative periods, the total dose of intraoperative rescue analgesia (fentanyl), and the level of sedation during the postoperative period, according to group allocation. Additional outcomes included intraoperative anaesthetic requirements (propofol, isoflurane), the physiologic parameters (HR, RR, non-invasive BP) recorded at predefined timepoints (T_0_–T_5_), pre- and postoperative serum total magnesium concentrations, the occurrence of adverse effects potentially related to magnesium administration (salivation, nausea, vomiting, bradycardia, arrhythmias, hypotension, or apnoea during induction), the need for mechanical ventilation, patellar reflex assessment up to 4 h postoperatively, and the recovery quality. Patellar reflexes were evaluated to assess muscle relaxation relative to magnesium administration, as described by Saberfard et al. (36), with dogs in lateral recumbency and scored as normal (+2) or abnormal (0 or +1, corresponding to absent or reduced reflex, respectively). This assessment was included as a practical clinical indicator of potential magnesium-associated neuromuscular effects, as it represents a reliable spinal reflex in dogs, and reduction or loss of deep tendon reflexes is a recognized early manifestation of hypermagnesaemia. Recovery quality was classified as acceptable (ideal, smooth and uncomplicated without vocalization) or unacceptable (difficult or rough with vocalization or dysphoria), according to a previous study (37).

Finally, demographic variables, surgery and anaesthesia duration, extubation time and time to first head movement, were recorded in minutes. Surgery duration was defined as the interval from the first skin incision to the final suture placement and skin closure, while anaesthesia duration was the time from induction to the discontinuation of isoflurane administration. Extubation time was defined as the interval from termination of isoflurane to removal of the endotracheal tube with return of the swallowing reflex, and time to first head movement was defined as the interval from isoflurane discontinuation to the first observed voluntary and purposeful head movement.

#### 2.6.1 Pain and sedation assessment

Pain and sedation were assessed preoperatively (baseline, before premedication), and at 1, 2, 4, 8, 12, 18, 24, 36 and 48 h after extubation. All assessments were performed by the same blinded evaluator, experienced in the respective scoring systems and unaware of treatment allocation. Sedation and pain were assessed in the same sequence at each timepoint, with a 1–2-min interval between each scoring. Initially, the level of sedation was evaluated using a composite, validated scale (score range 0–21), designed to assess the degree of sedation in dogs (38). Pain was assessed using the CMPS-SF (scores 0–24). The respective pain scoring system is a validated tool for the assessment of acute pain in dogs and consists of six behaviour-related categories. The maximum pain score that can be recorded is 24, indicating the highest level of pain intensity (31). Buprenorphine 10 μg kg^−1^ IV was administered as rescue analgesia if scores were ≥ 6/24. Pain was reassessed 30 min after rescue and dogs that received rescue analgesia were excluded from further statistical analysis; however, all dogs were assessed for apparent signs of pain until discharge, at 48 h after extubation.

#### 2.6.2 Total magnesium concentration measurement

Venous blood samples were collected from the jugular vein of all dogs preoperatively (during routine evaluation and bloodwork) and immediately after surgery (at the end of intraoperative magnesium sulphate or saline administration, according to group allocation) to measure serum total magnesium concentrations (mg/dL), using a veterinary analyzer (IDEXX Catalyst ONE Chemistry Analyzer, IDEXX Laboratories, USA).

#### 2.6.3 Adverse effects

The incidence of adverse effects was recorded throughout the perioperative period. Occurrence of clinical signs potentially related to magnesium sulphate administration included nausea, vomiting, hypersalivation, regurgitation, seizure activity, cardiovascular events (bradycardia, arrhythmia, hypotension) or respiratory depression (apnoea during induction or intraoperatively) was monitored throughout the perioperative period. Bradycardia and hypotension were defined as HR < 60 beats/min or mean BP < 60 mmHg for more than 5 min. The requirement for mechanical ventilation (if PETCO_2_ > 50 mmHg) during surgery and assessment of muscle relaxation (patellar reflex for the 4 postoperative hours) could potentially be related to magnesium sulphate administration as well; however, they were separately documented for statistical comparison between groups.

### 2.7 Sample size calculation

Before data collection an *a priori* power analysis was conducted using G*Power (version 3.1.9.7), to determine the minimum required sample size. A two-tailed independent-samples *t*-test was specified, with an *α*-level of 0.05 (type I error) and a desired power of 0.8. A between-group difference of 2.0 points on the CMPS-SF with a standard deviation (SD) of 1.8, was considered clinically relevant based on the original validation of the scale (39), corresponding to a large effect size (Cohen’s *d* = 1.11). Under these parameters, it was calculated that a total of 28 dogs (14 per group) was required to achieve 80.8% power to detect the specified difference at the 5% significance level.

The 4-h postoperative timepoint was chosen as a reference for the calculation, as previous studies indicate that pain intensity typically peaks within 1–4 h after OVH, and rescue analgesia is most likely required during this period (40). However, this choice does not restrict the analysis to 4 h, and all timepoints were evaluated to assess the duration of analgesic efficacy.

### 2.8 Statistical analysis

Statistical analysis was performed using IBM SPSS Statistics 29. Nominal variables were summarized as frequencies and percentages, and ordinal variables as frequencies and cumulative frequencies. Continuous variables are presented as mean ± SD. The normality of data distribution, including pain and sedation scores, was assessed using the Shapiro–Wilk test due to the relatively small sample size (<30). In all cases, normality was not rejected; therefore, parametric tests were applied. Between-group comparisons of continuous, normally distributed variables (e.g., age, weight, surgery and anaesthesia duration, extubation time, time to first postoperative head movement, total induction propofol dose, total intraoperative fentanyl dose) were performed using the independent-samples *t*-test. Categorical variables (e.g., number of dogs requiring rescue analgesia, incidence of adverse effects, requirement for mechanical ventilation, patellar reflex assessment, quality of recovery assessment) were compared using the Chi-square or Fisher’s exact test, as appropriate. Paired observations, such as pre- and postoperative magnesium concentrations within groups, were analyzed using the paired-samples *t*-test. For independent variables with more than two levels, one-way ANOVA was applied. Repeated intraoperative measurements, including HR, RR, BP and isoflurane requirements, and postoperative pain and sedation scores, were analyzed using repeated-measures ANOVA, to assess differences between groups over time, with Bonferroni correction for post-hoc comparisons where appropriate. Correlations between continuous variables (e.g., magnesium concentrations, analgesic requirements, pain scores) were assessed using Pearson’s correlation coefficient, while correlations involving ordinal variables were analyzed using Spearman’s rank correlation coefficient. All statistical tests were two-tailed and a *p*-value < 0.05 was considered statistically significant.

## 3 Results

### 3.1 Study population

All dogs enrolled in the study (*n* = 28) were included in the analysis, and no exclusions occurred. Complete datasets were available for all dogs for cardiovascular variables, BP measurements and serum magnesium concentrations; no data were missing. Groups did not differ significantly with regard to age, body weight, surgery duration and anaesthesia duration (Table 1).

**Table 1:** Age, weight, surgery duration, anaesthesia duration, extubation time and time to first postoperative head movement (mean ± SD) in dogs undergoing OVH.

Data	Magnesium (*N* = 14)	Control (*N* = 14)	*p*-value
Age (months)	26.3 ± 14.1	28.3 ± 14.7	0.727
Weight (kg)	13.8 ± 7.4	17.2 ± 9.0	0.314
Surgery duration (minutes)	33.7 ± 2.5	35.8 ± 3.0	0.060
Anaesthesia duration (minutes)	51.9 ± 3.5	54.5 ± 4.0	0.085
Extubation time (minutes)	5.3 ± 1.3^*^	3.3 ± 1.2	0.000
First postoperative head movement (minutes)	7.4 ± 1.9	4.8 ± 2.0	0.002

Dogs in the magnesium group (*n* = 14) received a 50 mg/kg IV bolus of magnesium sulphate followed by a continuous infusion of 20 mg/kg/h throughout surgery. Dogs in the Control group (*n* = 14) received an equivalent volume of 0.9% NaCl solution using the same timing and infusion protocol.

### 3.2 Intraoperative period

#### 3.2.1 Analgesic and anaesthetic requirements

The total dose of intraoperative rescue fentanyl was 0.3 ± 0.6 μg kg^−1^ in the Magnesium group and 0.6 ± 0.8 μg kg^−1^ in the Control group, with the difference being insignificant (*p* = 0.275). Fewer dogs in the Magnesium group required rescue analgesia intraoperatively; however, this difference was also insignificant (3/14, 21.4% vs. 6/14, 42.8%; *χ*^2^ = 0.680, *p* = 0.411).

**Figure 2:**
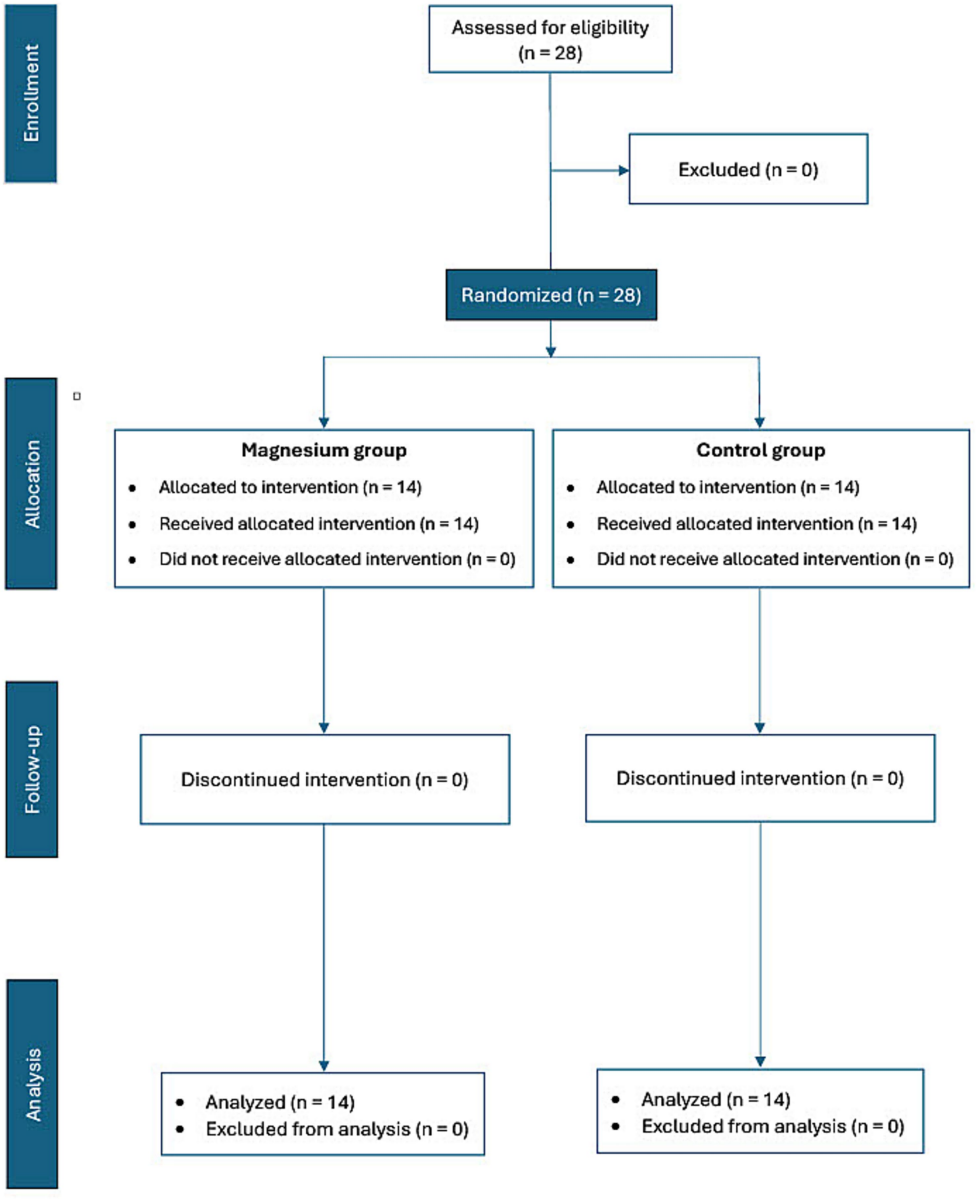
The flow chart of the study.

The mean propofol induction dose was significantly lower in the Magnesium group compared with the Control group (2.8 ± 0.5 mg kg^−1^ vs. 3.7 ± 0.4 mg kg^−1^; *p* < 0.01) (Figure 2). Dogs in the Magnesium group required 24% lower propofol for induction of anaesthesia, compared with the Control group.

Isoflurane end-tidal concentrations in dogs undergoing OVH did not differ between groups at any intraoperative timepoint (Table 2). End-tidal isoflurane concentrations varied over time (*F* = 133.156, *p* < 0.01); however, no significant interaction was observed between time and treatment group (*F* = 5.067, *p* = 0.112).

**Table 2:** Mean values, SD and level of significance of end-tidal isoflurane concentrations at different intraoperative timepoints (T_0_, T_1_, T_2_, T_3_, T_4_, T_5_) in Magnesium and Control groups in dogs undergoing OVH.

Variable	Group	*N*	Mean	SD	*p*-value
FE_ISO_ T_0_ (%)	Magnesium	14	1.3	0.0	1.000
	Control	14	1.3	0.0	
FE_ISO_ T_1_ (%)	Magnesium	14	1.3	0.0	0.327
	Control	14	1.3	0.0	
FE_ISO_ T_2_ (%)	Magnesium	14	1.3	0.0	0.060
	Control	14	1.4	0.1	
FE_ISO_ T_3_ (%)	Magnesium	14	1.4	0.0	0.659
	Control	14	1.4	0.0	
FE_ISO_ T_4_ (%)	Magnesium	14	1.4	0.0	0.089
	Control	14	1.5	0.1	
FE_ISO_ T_5_ (%)	Magnesium	14	1.5	0.1	0.090
	Control	14	1.5	0.1	

#### 3.2.2 Physiologic variables

The mean HR, and systolic, mean and diastolic BP values were significantly different at timepoints T_0_ and T_1_, but no differences were observed at the remaining intraoperative timepoints (T_2_, T_3_, T_4_, T_5_). The mean values of HR, and systolic, mean and diastolic BP values were significantly lower in the Magnesium group compared with the Control group at timepoints T_0_ (*p* = 0.027; *p* = 0.040; *p* = 0.011; *p* = 0.007, respectively) and T_1_ (*p* = 0.040; *p* = 0.047; *p* = 0.004; *p* = 0.007, respectively) (Table 3).

**Table 3:** Mean ± SD values of HR, and systolic, mean and diastolic BP in Magnesium and Control groups in dogs undergoing OVH at specific intraoperative timepoints (T_0_, T_1_, T_2_, T_3_, T_4_, T_5_).

**Variable**	**Group**	***N***	**T**_**0**_	**T**_**1**_	**T**_**2**_	**T**_**3**_	**T**_**4**_	**T**_**5**_
Heart rate (beats/min)	Magnesium	14	76.4 ± 11.6^*^	79.5 ± 12.2^*^	85.4 ± 16.0	81.7 ± 13.7	81.9 ± 13.8	82.3 ± 12.3
	Control	14	86.2 ± 9.5	89.5 ± 11.2	96.2 ± 14.6	88.0 ± 10.8	87.1 ± 10.5	84.6 ± 10.7
Systolic BP (mmHg)	Magnesium	14	105.7 ± 7.3^*^	107.8 ± 12.1^*^	124.5 ± 19.9	115.0 ± 13.8	110.3 ± 14.4	107.2 ± 11.2
	Control	14	113.7 ± 11.1	116.3 ± 9.5	130.5 ± 21.1	118.1 ± 17.8	116.8 ± 12.0	115.9 ± 12.2
Mean BP (mmHg)	Magnesium	14	77.6 ± 4.5^*^	76.8 ± 7.8^*^	91.8 ± 18.1	83.1 ± 8.5	80.9 ± 9.2	78.0 ± 8.7
	Control	14	85.6 ± 9.5	86.4 ± 7.7	96.2 ± 15.7	89.4 ± 11.0	86.5 ± 5.9	84.0 ± 7.5
Diastolic BP (mmHg)	Magnesium	14	60.8 ± 6.6^*^	60.3 ± 7.1^*^	71.9 ± 12.0	68.7 ± 9.6	65.4 ± 8.8	61.8 ± 7.4
	Control	14	70.5 ± 9.9	69.7 ± 9.0	79.2 ± 16.1	74.7 ± 8.9	71.3 ± 7.8	65.5 ± 8.9

^*^Indicates a value significantly lower than the corresponding value in the Control group at the same timepoint (*p* < 0.05).

More specifically, mean HR varied over time (*F* = 9.962, *p* < 0.01), and a significant interaction was observed between time and treatment group (*F* = 2.819, *p* = 0.019). Mean BP (systolic, mean and diastolic) values also varied over time (*F* = 8.715, p < 0.01; *F* = 9.523, p < 0.01; *F* = 8.459, p < 0.01, respectively), however, no significant interaction was observed between time and treatment group (*F* = 0.237, *p* = 0.946; *F* = 0.412, *p* = 0.840; *F* = 0.201, *p* = 0.962).

No bradycardia (HR < 60 beats/min) or hypotension (mean BP < 60 mmHg) was observed in any dog in either the Magnesium or the Control group.

The mean RR values were not significantly different between groups throughout anaesthesia. Mean RR values varied over time (*F* = 12.547, *p* < 0.01); however, no significant interaction was observed between time and treatment group (*F* = 0.340, *p* = 0.888). Values represent spontaneous RR, and dogs receiving IPPV were excluded from RR analysis at the corresponding timepoints (Table 4).

**Table 4:** Mean ± SD values of RR (breaths/min) in Magnesium and Control groups in dogs undergoing OVH at specific intraoperative timepoints (T_0_, T_1_, T_2_, T_3_, T_4_, T_5_).

Variable	Group	*N*	Mean	SD	*p*-value
Respiratory rate T_0_ (breaths/min)	Magnesium	14	10.4	1.4	0.820
	Control	14	10.2	2.0	
Respiratory rate T_1_ (breaths/min)	Magnesium	12	11.4	1.7	0.439
	Control	13	10.8	2.3	
Respiratory rate T_2_ (breaths/min)	Magnesium	10	13.8	3.8	0.837
	Control	12	13.5	3.7	
Respiratory rate T_3_ (breaths/min)	Magnesium	8	11.2	1.8	0.653
	Control	10	11.6	2.5	
Respiratory rate T_4_ (breaths/min)	Magnesium	7	11.1	1.5	0.540
	Control	8	10.6	2.2	
Respiratory rate T_5_ (breaths/min)	Magnesium	7	10.3	1.3	0.667
	Control	8	10.6	2.2	

Values represent spontaneous RR. Dogs that received intermittent positive pressure ventilation (IPPV) were excluded from RR analysis at the corresponding timepoints; the number (*N*) of dogs not requiring IPPV is indicated in the respective column in the table.

#### 3.2.3 Intraoperative mechanical ventilation requirement

Seven of 14 dogs (50%) in the Magnesium group and six of 14 dogs (42.9%) in the Control group required IPPV intraoperatively, with no significant difference between groups (*p* = 0.722) (Table 5).

**Table 5:** Prevalence of postoperative rescue analgesia and intraoperative mechanical ventilation requirement, adverse effects, recovery quality and patellar reflex assessment in Magnesium and Control groups.

Parameter	Classification	Magnesium (*N* = 14)		Control (*N* = 14)		p-value
		*Ν*	%	*Ν*	%	
Postoperative rescue analgesia	No	11	78.6%	9	64.3%	0.673
	Yes	3	21.4%	5	35.7%	
Intraoperative mechanical ventilation requirement	No	7	50%	8	57.1%	0.722
	Yes	7	50%	6	42.9%	
Adverse effects	No	9	64.3%	11	78.6%	0.683
	Yes	5	35.7%	3	21.4%	
Quality of recovery	Unacceptable	1	7.1%	4	28.6%	0.322
	Acceptable	13	92.9%	10	71.4%	
Patellar reflex	Abnormal	4	28.6	0	0%	0.096
	Normal	10	71.4%	14	100%	

Rescue analgesia, intraoperative mechanical ventilation requirement and adverse effects were recorded as yes/no. Recovery quality was classified as acceptable/unacceptable. Patellar reflex was classified as normal/abnormal.

### 3.3 Postoperative period

Extubation time and time to first postoperative head movement were significantly longer in the Magnesium group compared to the Control group (*p* < 0.01) (Table 1).

#### 3.3.1 CMPS-SF scores (primary outcome)

**Figure 3:**
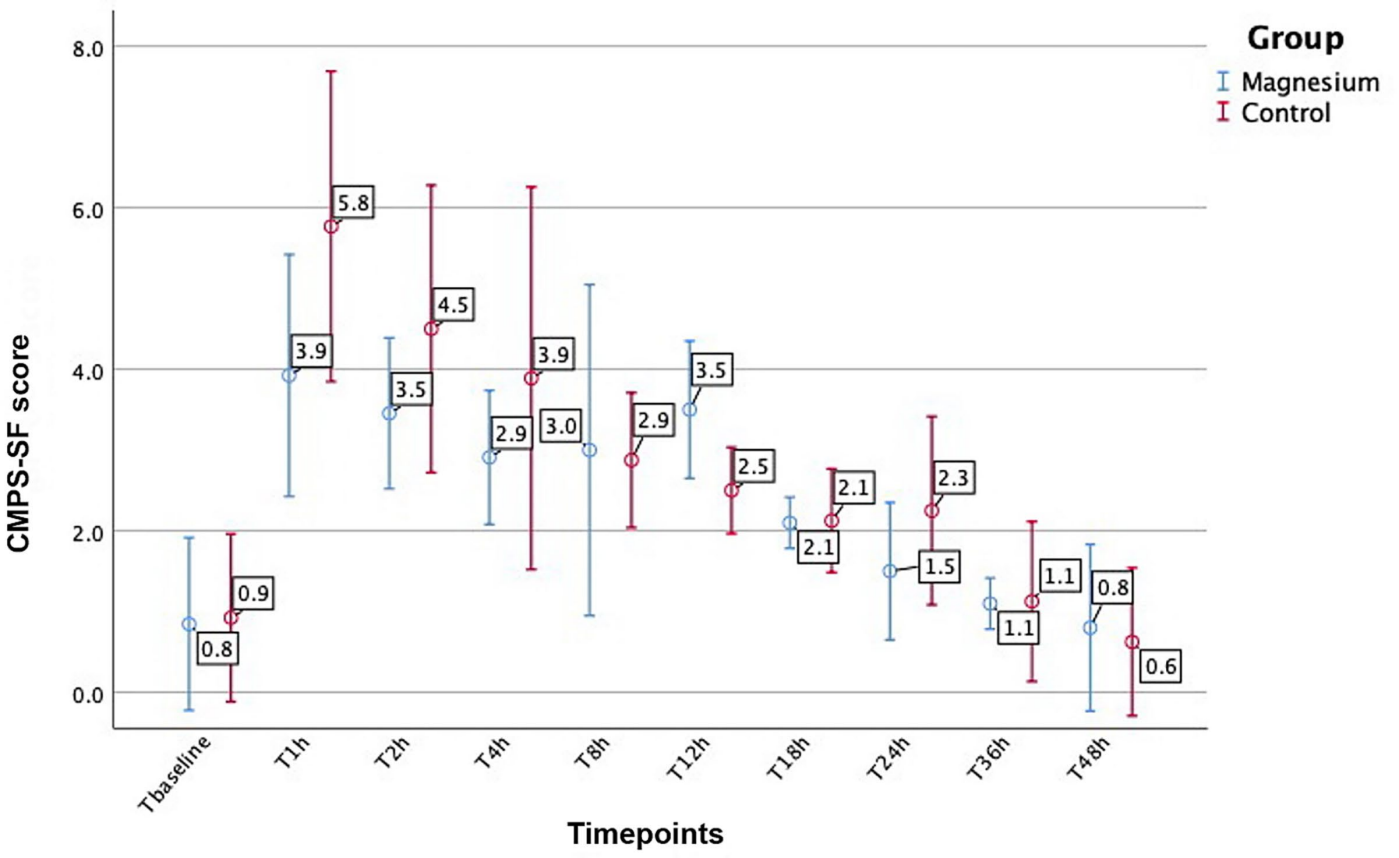
Mean baseline CMPS-SF scores and scores over the 9 postoperative time points.

Mean CMPS-SF scores were significantly lower in the Magnesium group compared with the Control group, 1 h after extubation (*p* = 0.012) (31). Pain scores were lower in the Magnesium group at 2 and 4 postoperative hours; however, these differences were not significant (*p* = 0.104 and *p* = 0.215, respectively). Mean CMPS-SF scores varied over time (*F* = 72.368, p < 0.01), and a significant interaction was observed between time and treatment group (*F* = 2.851, *p* = 0.004). Mean baseline CMPS-SF scores and scores over the 9 postoperative timepoints are presented in Figure 3.

#### 3.3.2 Sedation scores

Sedation scores did not differ between groups at any postoperative timepoint. Mean sedation scores varied over time (*F* = 250.954, *p* < 0.01); however, no significant interaction was observed between time and treatment group (*F* = 0.571, *p* = 0.722) (38).

#### 3.3.3 Rescue analgesia requirements

Three dogs (21.4%) in the Magnesium group and five dogs (35.7%) in the Control group required rescue analgesia throughout the postoperative period, with no significant difference between groups (*χ*^2^ = 0.181, *p* = 0.673) (Table 5).

One dog in the Magnesium group and three dogs in the Control group required rescue analgesia at 1 h postoperatively with this difference also being insignificant (*χ*^2^ = 0.295, *p* = 0.587). Until 4 h postoperatively, two dogs required rescue analgesia in the Magnesium group and five dogs in the Control group, but this difference was not deemed significant, either (*χ*^2^ = 0.782, *p* = 0.377). The remaining dog in the Magnesium group that required rescue analgesia did so at 8 h postoperatively.

#### 3.3.4 Quality of recovery

One of 14 dogs (7.1%) in the Magnesium group exhibited unacceptable recovery, compared with four of 14 dogs (28.6%) in the Control group. This difference was not significant (*χ*^2^ = 0.990, *p* = 0.322) (Table 5).

#### 3.3.5 Patellar reflex assessment

The patellar reflex was assessed in both groups until 4 h postoperatively and was classified as normal or abnormal (reduced). A reduced patellar reflex was observed in 4 of 14 dogs (28.6%) in the Magnesium group, whereas all dogs in the Control group exhibited normal patellar reflexes; this difference was not significant (*χ*^2^ = 2.659, *p* = 0.096) (Table 5).

The reduced patellar reflex in the Magnesium group was observed at 1 h post-extubation in all affected dogs, and normal deep tendon reflexes were restored in all four dogs by the 2-h postoperative assessment.

### 3.4 Perioperative adverse effects

In the Magnesium group, five of 14 dogs (35.7%) exhibited adverse effects, whereas three of 14 dogs (21.4%) in the Control group did so. There was no significant difference between groups in the incidence of the predefined adverse effects (*χ*^2^ = 0.191, *p* = 0.683) (Table 5).

In the Magnesium group, hypersalivation occurred in 3 of the 5 dogs exhibiting adverse effects, while the remaining two dogs experienced transient apnoea during the induction phase. Two of the three dogs with hypersalivation during induction also vomited. In the Control group, one dog exhibited hypersalivation 1 h post-extubation and vomited at 2 h post-extubation, one dog vomited 24 h postoperatively, and the third dog experienced transient apnoea during the induction phase. All episodes of nausea or vomiting in both groups were transient and resolved spontaneously; no dogs required anti-nausea or antiemetic medication postoperatively.

### 3.5 Serum total magnesium concentrations

The mean total serum magnesium concentrations in postoperative samples were 80% higher than the preoperative baseline values in the Magnesium group (*p* < 0.01). The corresponding difference between preoperative and postoperative magnesium concentrations in the Control group was not significant (*p* = 0.268).

Baseline magnesium concentrations did not differ between the Magnesium and Control groups; however, postoperative concentrations were significantly higher in the Magnesium group compared with the Control group (*p* < 0.01) (Table 6).

**Table 6:** Minimum, maximum, mean and SD of total serum magnesium concentrations in preoperative (baseline) and postoperative blood samples in the Magnesium and Control groups.

	Magnesium				Control			
	Minimum	Mean	SD	Maximum	Minimum	Mean	SD	Maximum
Mg baseline (mg/dL)	1.78	2.01	0.11	2.18	1.88	2.02	0.09	2.24
Mg postop (mg/dL)	3.02	3.64^*^	0.53	4.80	1.87	2.03	0.09	2.22

^*^Indicates a value significantly higher compared with baseline in the Magnesium group (*p* < 0.05). Furthermore, it indicates a value significantly higher than the corresponding value in the Control group at the same timepoint (*p* < 0.05). Normal total serum magnesium values: 1.40–2.38 mg/dL.

## 4 Discussion

Our findings indicate that intraoperative magnesium sulphate administration could result in a reduction in acute postoperative pain intensity 1 h after extubation in healthy dogs submitted to OVH. Additionally, magnesium administration just before the induction of anaesthesia contributed to a 24% reduction in propofol requirements for intubation of the trachea, however, no isoflurane-sparing effect was demonstrated. The significantly higher total serum magnesium postoperative concentrations in the magnesium group resulted in a prolonged recovery, however no other potential magnesium-related adverse effects were observed, suggesting that the current dose regimen is safe.

In the current study, buprenorphine, meloxicam and paracetamol were administered throughout the perioperative period, as part of a multimodal strategy, in accordance with strong recommendations for soft tissue procedures, and specifically OVH, in dogs (41). To further support an opioid-sparing approach, we incorporated a non-opioid agent such as magnesium sulphate into this regimen, given the ongoing interest in identifying safe adjunct analgesics that may potentially reduce perioperative opioid requirements. Considering the absence of established dosing recommendations for magnesium as an analgesic in dogs, with previously used doses largely extrapolated from human medicine, and given that earlier canine studies have not demonstrated a clear clinical analgesic benefit, we elected to use the highest recommended dose for humans (50 mg kg^−1^ loading bolus followed by a continuous infusion of 20 mg kg^−1^ h^−1^). This dosing strategy has not been previously evaluated in dogs; therefore, the present study helps address this knowledge gap by providing insights into both the safety and potential efficacy of this regimen in veterinary clinical practice.

### 4.1 Intraoperative period

#### 4.1.1 Analgesic—anaesthetic requirements

The number of dogs requiring rescue analgesia intraoperatively and the total fentanyl dose for rescue analgesia were both lower in the Magnesium group compared with the Control group; however, these differences were not statistically significant.

In human anaesthesia, adjunctive IV magnesium sulphate has frequently been associated with reduced intraoperative opioid requirements. Early studies in spinal surgery demonstrated that a loading dose of 30 mg kg^−1^ magnesium followed by 10 mg kg^−1^ h^−1^ decreased intraoperative remifentanil and fentanyl consumption (42, 43). A 2017 systematic review and meta-analysis of 13 studies (694 patients) reported that most trials using magnesium doses of 30–50 mg kg^−1^ followed by 6–25 mg kg^−1^ h^−1^ showed reduced intraoperative opioid use, although four studies found no difference (44). These four studies found no difference in intraoperative opioid consumption between magnesium-treated and control patients, whereas studies in which fentanyl was used as rescue analgesia during surgery reported lower requirements in the magnesium groups (44). More recent evidence remains inconsistent; remifentanil requirements were reduced during lumbar laminectomy using a 20 mg kg^−1^ loading dose followed by 20 mg kg^−1^ h^−1^ of magnesium infusion (45), whereas thoracotomy patients receiving 40 mg kg^−1^ followed by 10 mg kg^−1^ h^−1^ showed no significant differences (46).

In veterinary literature, no definite conclusions can be drawn regarding the effect of intraoperative IV magnesium on intraoperative analgesic requirements. Anagnostou et al. (17) observed that no dog included in their study of dogs undergoing OVH required any rescue fentanyl intraoperative intervention, between dogs receiving magnesium sulphate (50 mg kg^−1^ IV followed by a continuous infusion of 12 mg kg^−1^ h^−1^) and controls. The discrepancy from our findings, where six dogs in the Control group required rescue fentanyl intraoperative compared to three dogs in the Magnesium group with no significant difference, may largely reflect differences in study design. In Anagnostou et al. (17) intraoperative analgesia was not a primary outcome; depth of anaesthesia was adjusted primarily via halothane concentration based on cardiovascular, respiratory and neurological variables, and only dogs failing to respond to increased anaesthetic depth received fentanyl and were subsequently excluded. Such criteria may confound nociceptive responses with anaesthetic depth, as autonomic changes under surgical anaesthesia may be more correlated to inadequate analgesia rather than insufficient hypnosis (47–49). In contrast, in our study all dogs commenced surgery at a standardized surgical depth of anaesthesia (target end-tidal isoflurane concentration of 1.3%, corresponding to determined MAC in dogs (33), along with consistent reflex assessment), and acute or abrupt increases in physiologic parameters were specifically treated as indicators of inadequate analgesia, prompting rescue fentanyl administration. Thus, the methodological differences between studies limit direct comparison of intraoperative analgesic requirements. Another clinical study investigating the effect of intraoperative IV magnesium administration (50 mg kg^−1^ IV followed by a 15 mg kg^−1^ h^−1^ infusion) on postoperative analgesia, did not report intraoperative analgesic requirements (19), whereas a more recent study reported reduced intraoperative rescue requirements after a 50 mg kg^−1^ IV loading bolus followed by an intraoperative infusion of 80 mg kg^−1^ h^−1^ (27). Reduced opioid requirements have been also demonstrated in OVH studies in which magnesium was administered intrathecally (21) or intraperitoneally (24), rather than IV.

Regarding the effect of magnesium on anaesthetic requirements, our study showed that IV magnesium administration (50 mg kg^−1^ loading dose followed by a 20 mg kg^−1^ h^−1^ infusion) resulted in a 24% reduction in propofol requirements for induction. However, no corresponding reduction in isoflurane requirements during maintenance of anaesthesia was observed.

Evidence of magnesium anaesthetic-sparing properties in human medicine dates back to the early 20th century, when its administration was reported to “abolish pain sensation and consciousness in humans” (50). More recent human clinical data support this effect. A systematic review and meta-analysis demonstrated that administering 30–50 mg kg^−1^ IV magnesium at least 10 min before induction significantly reduced propofol induction doses in six studies including 320 human patients (44). Consistent with these findings, in our study a 50 mg kg^−1^ IV loading dose administered approximately 15 min before induction resulted in a 24% reduction in propofol requirements. Similar induction-sparing effects have been reported in other studies in dogs. A recent blinded, randomized trial found that propofol requirements were 31.2% lower after a 50 mg kg^−1^ bolus followed by 30 mg kg^−1^ h^−1^ infusion, and 38.9% lower when followed by 80 mg kg^−1^ h^−1^ (26). Anagnostou et al. (17) also reported an 11.9% reduction in thiopental requirements after the same bolus dose. Magnesium appears to reduce anaesthetic requirements during maintenance, as well. In humans, loading doses of 30–50 mg kg^−1^ followed by infusions of 8–20 mg kg^−1^ h^−1^ have significantly decreased propofol consumption (43, 44, 51). In dogs, reductions in propofol maintenance requirements have been reported after a 50 mg kg^−1^ bolus followed by infusions of 10 mg kg^−1^ h^−1^ (18) and 80 mg kg^−1^ h^−1^ (26).

Magnesium’s anaesthetic-sparing effects have also been demonstrated with volatile agents in humans, where doses of 30–40 mg kg^−1^ followed by a 10 mg kg^−1^ h^−1^ infusion reduced intraoperative desflurane requirements (52, 53). Findings in dogs, however, are inconsistent. Anagnostou et al. (17) reported an approximately 20% reduction in halothane requirements after a 50 mg kg^−1^ bolus followed by a 12 mg kg^−1^ h^−1^ infusion, whereas other studies found no effect on isoflurane (19) or sevoflurane (22) maintenance requirements. Our results align with these latter studies, as no isoflurane-sparing effect for maintenance of anaesthesia was observed during magnesium infusion. End-tidal isoflurane concentrations in our study (mean 1.37% in the Magnesium group vs. 1.4% in the Control group) were similar to those reported by Rioja et al. (19), though slightly higher, likely due to our use of a predefined target concentration of 1.3% for at least 5–10 min before incision, as has been determined for MAC in dogs (33). In contrast, isoflurane in the Rioja et al. (19) study was titrated solely by reflex assessment. Differences between our findings and human studies demonstrating reduced desflurane requirements (52, 53) may be explained by the method of anaesthetic depth assessment. Human trials used BIS monitoring (target values of 45–55 and 40–60, respectively, which account for a surgical anaesthetic depth), providing an objective measure and allowing precise titration of volatile anaesthetics, whereas veterinary studies, including ours, relied on subjective clinical indicators of anaesthetic depth.

#### 4.1.2 Physiologic variables

Regarding intraoperative physiologic monitoring, no episodes of hypotension (MAP<60 mmHg) or bradycardia (HR < 60 beats/min) were observed, whereas no difference in respiratory depression and need for mechanical ventilation was reported in either group of our study. Although iatrogenic hypermagnesaemia has been associated with bradycardia, impaired respiration, or mild to moderate hypotension (3, 5), dogs receiving magnesium in our study exhibited only modest reductions in systolic, mean and diastolic arterial BP at the early intraoperative timepoints (T_0_, T_1_). These changes were not clinically significant as all values remained within predefined normal limits and required no intervention. Mean BP values varied over time but showed no significant time-treatment interaction. HR followed a similar pattern: no clinically relevant bradycardia occurred (HR < 60 beats/min), though HR was consistently lower in the Magnesium group, particularly at T_0_, T_1_, where differences reached statistical significance. This is likely to reflect the proximity of these timepoints to the initial 50 mg kg^−1^ bolus, given the reported magnesium half-life of approximately 13 min in dogs (54). Dose-dependent decreases in HR and BP following IV magnesium administration have previously been reported at 60–120 mg kg^−1^, however, without affecting cardiac output (55). The absence of notable haemodynamic disturbances is consistent with human studies using clinical doses of magnesium, despite transient postoperative hypermagnesaemia (6, 14–16, 52). Similar findings have been reported in canine studies using human-recommended dosing regimens, with no noteworthy magnesium-associated bradycardia, hypotension or respiratory depression observed (17–19, 26, 27). In our study, RR and the incidence of mechanical ventilation also did not differ between the Magnesium and Control groups, further supporting the cardiovascular and respiratory safety of the administered dose.

### 4.2 Postoperative period

Pain management is a fundamental component of successful surgical outcome in veterinary clinical practice, and routine perioperative pain assessment is essential for optimizing patient care (41, 56). In our study, pain and sedation levels were assessed by the same investigator at predefined timepoints from the preoperative period until 48 h post-extubation. Dogs receiving magnesium showed significantly lower pain scores at 1 h post-extubation. Although pain scores remained lower in the Magnesium group at 2 h (3.5 ± 0.9 vs. 4.5 ± 1.8 for Magnesium and Control groups, respectively) and 4 h (2.9 ± 0.8 vs. 3.9 ± 2.4 for Magnesium and Control groups, respectively), these differences were not statistically significant. The development and validation of pain scales have greatly improved reliability in companion animal pain assessment. Unidimensional tools, such as the NRS are simple to use but more subjective, whereas multidimensional scales based on behavioural variations provide more reliable evaluations (57). The CMPS-SF, validated for acute postoperative pain, incorporates spontaneous behaviour and animal-assessor interaction and can discriminate pain levels even when the evaluator is not a native English speaker (58), as was the case in our study. A correlation between unidimensional tools (such as the NRS) and CMPS-SF scores has been demonstrated especially when applied simultaneously; however, selecting the NRS as the primary scale over the use of the validated CMPS-SF, requires caution (59). Regarding postoperative rescue analgesia, 3 dogs (21.4%) in the Magnesium group and 5 dogs (35.7%) in the Control group required buprenorphine within 48 h, with no significant difference between groups. This aligns with previous findings indicating that dogs undergoing OVH are most likely to require rescue analgesia between 1 and 4 h postoperatively (40), which was also the case in our study.

Perioperative IV magnesium appears to be a useful adjunctive analgesic in humans undergoing general anaesthesia, reducing postoperative pain intensity and rescue analgesic requirements. Three meta-analyses reported significantly decreased opioid consumption during the first 24 h after surgery when magnesium was administered perioperatively (6, 14, 15), and two also demonstrated lower postoperative pain scores both at 4 h (15) and at 24 h (14, 15). In a 2020 study of 74 patients undergoing lumbar laminectomy with propofol-remifentanil TIVA, intraoperative magnesium significantly reduced pain scores (VAS) at all postoperative timepoints assessed (2, 4, 6 and 24 h) and decreased morphine consumption (45). Similarly, lower pain scores (NRS) and reduced rescue analgesia requirements were also reported when a ketamine-magnesium OFA protocol was compared with a remifentanil-based opioid anaesthetic approach (10). The analgesic benefits of magnesium may extend beyond immediate postoperative period. When magnesium infusion was continued for 20–24 h after surgery patients demonstrated lower NRS pain scores on postoperative days 1 and 8 and a reduced incidence of chronic neuropathic post-thoracotomy pain on days 30 and 80 (16, 46).

Regarding the three existing canine studies evaluating intraoperative IV magnesium administration as an analgesic, none demonstrated a beneficial effect on postoperative analgesic requirements or pain scores (17, 19, 27). Anagnostou et al. (17) and Boff et al. (27) reported no difference in rescue analgesia between dogs receiving magnesium sulphate (50 mg kg^−1^ IV bolus followed by 12 mg kg^−1^ h^−1^ or 80 mg kg^−1^ h^−1^, respectively) and the control group; however, postoperative pain assessment was not a primary outcome. Similarly, Rioja et al. (19) observed no improvement in postoperative pain scores after magnesium administration (50 mg kg^−1^ IV, followed by 15 mg kg^−1^ h^−1^), with both groups exhibiting comparably low pain scores. Differences in analgesic protocols may partly explain the discrepancy between these findings and our results, beyond differences in study design. Additionally, mechanistic considerations may also contribute to these variable findings. Preclinical and clinical evidence suggests that NMDA receptor antagonists may exert greater analgesic effects in chronic pain states than in acute nociceptive conditions, largely due to their role in modulating central sensitization. Sustained glutamatergic activation enhances NMDA receptor-mediated transmission in the dorsal horn, contributing to wind-up, long-term potentiation, neuronal hyperexcitability and the persistence of neuropathic and chronic pain conditions (2, 10, 11). In contrast, acute postoperative pain models such as OVH involve transient nociceptive stimulation, in which NMDA receptor activation may play a less dominant role. This pathophysiological distinction may partly contribute to the variable or sometimes less pronounced analgesic effects reported for magnesium in acute surgical settings compared with chronic pain conditions. Neither of the previous studies employed a preventive, multimodal analgesic strategy, and postoperative analgesia was not standardized, but administered according to pain scale assessments. In Anagnostou et al. (17), only a preoperative NSAID (carprofen) was used routinely, with postoperative pethidine or morphine administered based on VAS scores. In Rioja et al. (19), morphine was administered preoperatively to all dogs, but no additional intraoperative or postoperative analgesics were provided unless CMPS-SF scores guided rescue treatment with morphine or carprofen. This, however, does not imply inadequate analgesic management. Preoperative NSAID administration alone or combined with an opioid, can provide sufficient analgesia in dogs undergoing OVH (60–63). It is possible that these studies intentionally minimized analgesic confounders to detect a potential effect of magnesium, yet no clear analgesic benefit was demonstrated. In contrast, current WSAVA guidelines strongly recommend general anaesthesia combined with a preventive, multimodal analgesic approach for OVH, with postoperative analgesia continued for at least 3 days (41).

The concept of multimodal analgesia proposes that combining agents with different mechanisms or sites of action enhances analgesia, reduces opioid requirements and consequently decreases opioid-related adverse effects (64). In our study, a preventive multimodal protocol was used consisting of preoperative dexmedetomidine, buprenorphine and meloxicam, followed by postoperative paracetamol, in accordance with current WSAVA recommendations (41). Consistent with previous canine studies, the analgesic effects of magnesium on intraoperative and postoperative outcomes were modest compared with findings in human medicine. However, this topic remains substantially under-investigated in veterinary patients, whereas plethora of RCTs, systematic reviews and meta-analyses have evaluated perioperative IV magnesium across a wide range of surgical procedures and pain intensities in humans.

The first factor that may have limited a much clearer analgesic effect of magnesium in our study was the use of a preventive multimodal analgesic regimen, consistent with WSAVA guidelines, which likely masked its modest adjunctive contribution. Magnesium possesses no primary analgesic activity and is considered a secondary or complementary analgesic that enhances the actions of more established pain medications (9). Thus, the perioperative administration of buprenorphine, meloxicam and paracetamol may have contributed to the low prevalence of rescue analgesia and the absence of further significant differences between group pain scores. Buprenorphine, a long-acting partial *μ*-agonist opioid, is recommended at 20 μg kg^−1^ combined with an NSAID for postoperative pain after OVH in dogs (41, 60–62). When used as the sole analgesic, however, it provides insufficient postoperative pain control, with nearly half of dogs requiring rescue analgesia 4–8 h after surgery (62, 65, 66), most commonly within the first 1–4 h (65, 66). Therefore, in our study, buprenorphine at 20 μg kg^−1^ was expected to provide analgesia for only 2–4 h postoperatively. Meloxicam was administered IV approximately 30 min before surgery to ensure perioperative analgesic coverage. Peak plasma concentrations occur 2–3 h after SC administration (63), and NSAIDs are most effective when given preoperatively, whereas adequate postoperative pain control following preoperative meloxicam has been reported in OVH and other soft-tissue procedures (60, 63, 67). The combination of an opioid and an NSAID is considered effective multimodal analgesia for OVH in dogs (41, 60–62), and in our study, paracetamol 10 mg kg^−1^ was administered at the end of surgery in both groups to provide analgesia once buprenorphine’s effect diminished. Paracetamol has demonstrated efficacy comparable to meloxicam for postoperative OVH pain when administered preventively and regularly postoperatively (68). Overall, the robust multimodal regimen used in this study was likely sufficient to minimize postoperative pain in both groups, thereby potentially limiting the detectability of a sustained and consistent magnesium analgesic contribution, beyond the early postoperative period.

Pain is a multidimensional phenomenon that requires prompt and adequate management, as it becomes more difficult to control once established. Evidence from the existing literature indicates that dogs in our study were appropriately managed, from an analgesic perspective, throughout the perioperative period. Between 1 and 4 h after extubation, buprenorphine and meloxicam (administered preoperatively) and paracetamol (administrated at the end of surgery), were all effective. Although pain scores were significantly lower in the Magnesium group at 1 h post-extubation, the overall effectiveness of the multimodal regimen, acting at multiple sites along the pain pathway, likely limited the ability to detect significant differences in rescue analgesia consumption or in pain scores at 2 and 4 h. Rescue buprenorphine was required in 2 dogs in the Magnesium group and 5 dogs in the Control group, within the first 4 h, but this difference was not significant. The significantly lower pain scores at 1 h may reflect the immediate but short-lived analgesic effect of IV magnesium, which has been reported to last approximately 30 min (2). A unimodal analgesic protocol might have revealed a more pronounced magnesium-related effect; however, the objective of the present study was to evaluate magnesium as an adjunct within a multimodal framework. Limiting perioperative analgesics to accentuate magnesium’s effect would have raised welfare and ethical concerns. It is possible that an opioid-free protocol or procedures associated with more severe postoperative pain, potentially leading to central sensitization or transition to long-term maladaptive pain states such as persistent postoperative pain, including mastectomy, orthopaedic surgery or limb amputation (41), might have yielded more distinct results. Additionally, continuation of magnesium administration as a postoperative continuous infusion could prolong its analgesic effects beyond the early postoperative period; however, this warrants further investigation.

A second factor that may have limited the observable analgesic effect of magnesium is the moderate postoperative pain typically associated with OVH in dogs (41, 61). Given the preventive multimodal regimen used in our study, OVH may not have produced sufficient postoperative pain to reveal a clinically relevant adjunctive effect of magnesium. Previous studies in dogs have demonstrated that preoperative administration of an NSAID alone (60) or in combination with an opioid provides effective analgesia for OVH and other abdominal procedures (60–63, 67). This was evident in earlier canine magnesium studies, as neither Anagnostou et al. (17), nor Rioja et al. (19), nor Boff et al. (27) demonstrated reductions in postoperative analgesic requirements or pain scores. In the study of Anagnostou et al. (17), dogs received carprofen as the sole analgesic and pain assessment relied solely on the VAS, which may lack sensitivity in detecting differences in multidimensional pain experiences, particularly in non-verbal patients, compared with validated composite pain scales such as the CMPS-SF (31, 58, 69). Rioja et al. (19) used the CMPS-SF but found no magnesium-related analgesic benefit, likely because morphine was administered preoperatively and the postoperative evaluation period was limited to 3 h. Given the short duration of magnesium’s IV analgesic effect, reported to be approximately 30 min (2), and the 4-h duration of morphine action, any magnesium effect was likely masked, as acknowledged by the authors. In our study, buprenorphine (a less efficacious but longer-acting opioid) was used, with its analgesic efficacy shown to be inferior to that of methadone for OVH (66). Another explanation for the lack of detectable analgesic differences is statistical power, being acknowledged by the authors, as pain assessment was not a primary outcome in the study by Rioja et al. (19), or Boff et al. (27). Furthermore, more invasive surgical procedures associated with greater tissue trauma and higher postoperative pain intensity may have allowed clearer discrimination of magnesium’s adjunctive effects. NMDA receptor activation is more pronounced in conditions involving intense, sustained, or pre-existing nociceptive input, such as highly invasive surgeries or chronic pain states (2, 10, 11). In contrast, OVH represents an acute postoperative pain model, and NMDA receptor contribution to pain processing may be less dominant compared to persistent or chronic pain conditions. This may have limited the observable analgesic effect of magnesium in the present study. OVH was selected as the nociceptive model because few veterinary studies have investigated perioperative IV magnesium (only three) and results remain inconclusive. OVH is a standardized procedure widely used for pain assessment research (70, 71), with predictable postoperative pain intensity (41, 72). Moreover, the dosing regimen (50 mg kg^−1^ loading dose followed by 20 mg kg^−1^ h^−1^ infusion), also falls within human recommendations (2). Standardization of surgical technique, magnesium dosing, and the use of a single surgeon and a single postoperative pain assessor in the current study, minimized variability and enhanced reliability. The findings of this study may inform future research involving more invasive procedures with greater potential for chronic pain development, cases with pre-existing NMDA receptor activation, or protocols employing alternative analgesic strategies such as opioid-free anaesthesia. Peripheral and central sensitization are considered key drivers of persistent postoperative pain, whereas central sensitization develops rapidly and plays a more substantial role than previously appreciated (64). Thus, the use of NMDA antagonists, including magnesium, remains of interest as part of multimodal analgesia. Nevertheless, there are no reports in dogs of OVH leading to chronic postoperative pain, and pain-related behavioural changes typically resolve within 24 h (72), consistent with guidelines recommending postoperative analgesia for up to 3 days (41).

Apart from pain assessment, the level of sedation was also evaluated at the same timepoints. Because pain scoring is inherently subjective and can be influenced by sedation (59), assessing sedation was necessary to ensure that it did not confound pain evaluation. In our study, sedation levels did not differ between groups, suggesting that magnesium did not influence postoperative sedation. The sedation scale used was validated and capable of discriminating sedation levels in dogs (38). Scores range from 0 to 21 with 0–2 representing little or no sedation, 4–11 moderate sedation and ≥13 heavy sedation. Only scores at 1 h post-extubation fell within the moderate sedation range (4.5 ± 0.9 and 4.2 ± 0.7 for Magnesium and Control groups, respectively). From 2 h onward, scores were < 3, corresponding to little or no sedation. Since sedation may interfere with early postoperative pain scoring (41, 58) we used a validated tool and initiated pain assessment at 1 h post-extubation, when sedation was expected to be minimal and most dogs were indeed standing and interactive towards the assessor. In our study dexmedetomidine was not reversed. First, reversal would have eliminated its analgesic effect (73), which was undesirable in the context of our multimodal, opioid-sparing approach. Second, the expected impact of dexmedetomidine on postoperative sedation was minimal because of its short half-life: approximately 27 min (0.46 h) after a prolonged 24-h infusion (74), and 39.6 min (0.66 h) after a single 10 μg kg^−1^ IV bolus (75). In that study, all dogs were standing 90 min after administration. In ours, anaesthesia lasted approximately 60 min (51.9 ± 3.5 min vs. 54.5 ± 4.0 min for Magnesium and Control groups, respectively), dexmedetomidine was administered 20 min before induction, and the first pain assessment was performed approximately 140 min after its administration. Given the dose used (4 μg kg^−1^), sedation duration was expected to be shorter than in the study of Kuusela et al. (75). Similarly, dogs receiving 20 μg kg^−1^ of medetomidine were able to walk at 150 min post-injection (76), which was consistent with the observations in our study. In a separate OVH study medetomidine was reversed, potentially due to a very short surgical duration (23 min), but atipamezole caused marked hypersalivation (66), an undesirable effect in the current study given hypersalivation’s potential association with magnesium administration. Therefore, it seems that postoperative sedation was unlikely to have been potentiated by magnesium. Magnesium infusion did, however, prolong recovery times. Extubation and first head movement times were 60 and 54% longer in the Magnesium compared to the Control group, respectively (5.3 ± 1.3 vs. 3.3 ± 1.1 min; 7.4 ± 1.9 vs. 4.8 ± 2.0 min, respectively). This delay is most likely attributable to magnesium administration, but not to the fact that dexmedetomidine was not reversed which was identical across groups. Similar delays have been reported in human patients receiving magnesium under propofol anaesthesia (42, 43). Canine studies, however, showed mixed findings. Magnesium prolonged anaesthesia under thiopental (18) correlated with our results, was associated with faster extubation under propofol TIVA (26), showed no difference under propofol (27) or sevoflurane anaesthesia (22), and was not evaluated in other studies (17, 19). Finally, recovery quality classified as acceptable or unacceptable according to a previous study (37), did not differ between groups consistent with previous canine studies reporting smooth, uneventful recoveries following magnesium administration (18, 19, 22).

### 4.3 Serum total magnesium concentrations, adverse effects and patellar reflex assessment

In the present study we aimed to identify potential adverse effects specifically attributable to magnesium administration, while distinguishing them from effects related to other perioperative analgesic or anaesthetic agents. Buprenorphine has been extensively evaluated in previous studies without relevant adverse effects being reported (61). It produces minimal cardiovascular or respiratory effects and has not been associated with nausea or vomiting, unlike pure *μ*-agonists such as morphine (61, 62, 77). Notably, the incidence of vomiting has been documented in 50–75% of dogs after morphine administration (78). Additionally, combining buprenorphine with an NSAID has not been associated with significant adverse reactions (61), and paracetamol is considered safe in dogs when administered at recommended doses (10–15 mg kg^−1^ every 8–12 h), with no serious side-effects reported (68). For clarity, the interpretation of adverse effects is presented in relation to serum magnesium concentrations, followed by evaluation of clinical signs and neuromuscular assessment.

Adverse effects were recorded throughout the preoperative, intraoperative and postoperative periods, including hypersalivation, nausea, vomiting, seizure activity and undesirable cardiovascular (bradycardia, arrhythmias, hypotension) or respiratory events (hypercapnia, apnoea and need for mechanical ventilation). Iatrogenic hypermagnesaemia has been associated with absent or decreased deep tendon reflexes, respiratory depression, mild to moderate hypotension, bradycardia, cardiac conduction disturbances, and in severe cases cardiac arrest (3, 5). Even mild elevations in serum magnesium may reduce deep tendon reflexes, while further clinical signs of toxicosis include vomiting, nausea, and hypersalivation (1, 4). Although respiratory depression and mechanical ventilation requirement, hypotension and bradycardia have all been reported as magnesium-related adverse effects, we evaluated mechanical ventilation requirement separately rather than include it within the general adverse-effect analysis. This decision was based on the fact that mechanical ventilation need is not magnesium-specific and may be required intraoperatively due to other anaesthetic or analgesic agents. Common complications during general anaesthesia include hypotension, hypoventilation, hypoxia, hypothermia and tachycardia or bradycardia, with hypotension and hypoventilation particularly associated with dose-dependent effects of inhalant anaesthetics (79). Serum magnesium levels were also measured, to verify the effects of the administered magnesium regimen. All dogs received identical premedication and perioperative analgesic protocols; therefore, adverse effects potentially related to these agents would be expected to be evenly distributed between groups and are unlikely to explain between-group differences. Consequently, observed differences were interpreted primarily in relation to magnesium administration and corresponding serum concentrations. Additionally, all episodes of nausea or vomiting were transient, resolved spontaneously, and did not require anti-nausea or antiemetic treatment in any dog.

In our study, an initial IV magnesium sulphate loading bolus of 50 mg kg^−1^ administered before anaesthesia induction, followed by a continuous intraoperative infusion of 20 mg kg^−1^ h^−1^ resulted in an 80% increase in serum magnesium concentrations, compared with preoperative baseline values. Postoperative serum magnesium concentrations exceeded the reference interval for dogs (1.40–2.38 mg/dL), reflecting the effect of the administered magnesium regimen. Similar increases following intraoperative magnesium administration have been reported in both human and canine studies, with dose-dependent elevations ranging from 35 to 74% in humans (6, 13, 51, 52, 80) and 17.6–71% in dogs (17–19, 22). The highest increases in canine studies, i.e., 70 and 71%, were reported after IV administration of 50 mg kg^−1^ followed by 15 mg kg^−1^ h^−1^ and 45 mg kg^−1^ followed by 15 mg kg^−1^ h^−1^ of magnesium sulphate, respectively (19, 22). The greater increase (80%) observed in our study is likely attributable to the higher infusion rate (20 mg kg^−1^ h^−1^ vs. 15 mg kg^−1^ h^−1^). However, lower magnesium doses produced less pronounced increases; Helal and El-Dahrawy (18) reported a 17.6% increase in magnesium concentrations after a single 50 mg kg^−1^ IV bolus and a 30.9% increase after a 50 mg kg^−1^ bolus followed by a 10 mg kg^−1^ h^−1^ infusion. Anagnostou et al. (17) reported a 58% increase following a 50 mg kg^−1^ bolus and 12 mg kg^−1^ h^−1^ infusion, although magnesium samples were obtained 10–15 min after infusion termination, likely underestimating peak values compared to studies, including ours, where sampling was performed immediately after infusion cessation. Serum magnesium concentrations seem to decline rapidly once administration stops in animals with normal renal function (4) and a plasma half-life of approximately 35 min has been reported after a 48-min infusion of 68 mg kg^−1^ (54), potentially playing a role in the magnesium concentrations reported in the study of Anagnostou et al. (17).

In humans, magnesium concentrations of 3–4 mg/dL, classified as mild hypermagnesaemia, have been associated with nausea, vomiting, lethargy, hyporeflexia, hypotension and 1st degree atrioventricular block, whereas more severe hypermagnesaemia (10–12 mg/dL) may result in flaccid paralysis, coma, respiratory depression and asystole (81). In our study, preoperative magnesium concentrations were 2.0 ± 0.1 mg/dL in the Magnesium group, increasing to 3.6 ± 0.5 mg/dL immediately after infusion termination. No significant differences were observed between groups regarding respiratory depression (requirements for mechanical ventilation), hypotension, bradycardia, reduced deep tendon reflexes (patellar reflex assessment) or other adverse effects, such as hypersalivation or vomiting. Two dogs exhibited magnesium concentrations > 4 mg/dL (4.68 και 4.80 mg/dL), and both exhibited adverse effects such as hypersalivation, vomiting and decreased patellar reflexes. Hypersalivation occurred during induction in both dogs, one of them vomited, whereas both exhibited reduced patellar reflexes at 1 h postoperatively. Nevertheless, no significant differences in nausea or vomiting were detected between groups. This aligns with findings from a 2020 meta-analysis of 683 patients (6) and a clinical study in dogs (19), both reporting no magnesium-related increase in nausea or vomiting. Helal and El-Dahrawy (18) and Anagnostou et al. (17) reported episodes of nausea, hypersalivation or vomiting following magnesium administration, though no mention is made on the potential significance of their results. Helal and El-Dahrawy (18) observed hypersalivation but not vomiting in 1/5 dogs of each magnesium group, 2 min after the magnesium bolus, while Anagnostou et al. (17) reported nausea in 6/27 dogs and vomiting in 2 of them, occurring 1–2 min after magnesium administration. However, the rate of the bolus administration may have played a role. Helal and El-Dahrawy (18) did not report infusion duration, and Anagnostou et al. (17) infused the 50 mg kg^−1^ magnesium bolus over approximately 5 min. In contrast, magnesium in our study was administered over approximately 15 min, consistent with recommendations in human clinical practice (45, 46, 80).

No differences in the predefined cardiovascular and respiratory adverse effects were detected, as has been also mentioned above (Section 4.1.2). No episodes of hypotension (mean BP < 60 mmHg), bradycardia (HR < 60 beats/min), arrhythmias or respiratory depression (i.e., mechanical ventilation requirements) were recorded. Although dogs in the Magnesium group exhibited lower systolic, mean, diastolic BP values, as well as lower HR values, these changes were not clinically significant and required no intervention. Based on our findings and existing literature, the magnesium dosing regimen used in this study (50 mg kg^−1^ bolus, followed by 20 mg kg^−1^ h^−1^ infusion) appears safe. Higher bolus doses (90–132.4 mg kg^−1^) have produced unpredictable clinical effects (54, 55). Following IV administration of 132.4 mg kg^−1^, one dog experienced fatal cardiac arrest with extremely elevated magnesium concentrations (approximately 51 mg/dL) (54). This dose is approximately 3 times higher than the initial bolus used in our study and about twice the total magnesium dose administered during the 1-h procedure (approximately 70 mg kg^−1^). The lethal magnesium concentration reported (approximately 51 mg/dL) was roughly 15 times higher than the post-infusion values observed in our study. Moreover, such doses substantially exceed those recommended in human literature. Clinical studies in humans have similarly reported an absence of significant haemodynamic instability when clinically relevant magnesium doses were administered, despite postoperative magnesium concentrations exceeding normal limits (6, 14–16, 52). Comparable findings have been described in canine studies, where administration of IV magnesium at human-recommended doses did not produce cardiovascular or respiratory complications attributable to hypermagnesaemia, such as arrhythmias, bradycardia or hypotension (17–19, 26, 27).

We also evaluated whether magnesium administration affected deep tendon reflexes by assessing the patellar reflex up to 4 h after extubation. For statistical clarity, reflexes were classified as normal (+2) or abnormal (0 or +1, indicating absent or decreased reflexes). Four dogs in the Magnesium group exhibited decreased patellar reflexes postoperatively, whereas no dog in the Control group did; however, this difference was not statistically significant (*p* = 0.096). All four affected dogs had diminished reflexes at the 1-h post-extubation assessment, but reflexes were normal in all dogs by the 2-h postoperative evaluation, even in those with magnesium concentrations >4 mg/dL. This transient reduction is consistent with known effects of hypermagnesaemia which can decrease or abolish deep tendon reflexes, even at mild elevations of serum magnesium (1, 3–5). In contrast, human clinical studies (42, 52) and most canine studies (17–19, 22) have not described postoperative magnesium-related muscle weakness or reflex depression, although Júnior et al. (26) noted a subjective magnesium-induced muscle relaxation effect based on intubation quality, without corresponding differences on the intubation scoring. In our study two of the four dogs with decreased patellar reflexes had magnesium concentrations exceeding 4 mg/dL (4.68 and 4.80 mg/dL), while the remaining two had concentrations of 3.32 and 3.81 mg/dL. These findings occurred approximately 1 h after infusion cessation and resolved by 2 h post-extubation. All dogs had normal renal function, which likely facilitated the potentially rapid decline in serum magnesium and restoration of normal deep tendon reflexes, a phenomenon previously described (4). This is further supported by the short elimination half-life of magnesium in dogs, approximately 13 min after 15.8 mg kg^−1^ bolus, and 35 min after a 68 mg kg^−1^, for 48-min infusion (54).

Although statistically significant differences were identified for propofol requirements and early postoperative pain scores, the overall clinical impact was modest. Magnesium did not reduce isoflurane or rescue opioid requirements and did not provide sustained analgesia beyond the early postoperative period. Therefore, its clinical utility appears limited to a potential adjunctive role rather than a primary analgesic strategy. The observed delay in extubation and transient reduction in patellar reflexes, although not clinically consequential, should be considered when selecting dosing regimens.

### 4.4 Methodologic strengths and limitations

This study has several methodological strengths regarding its design. All individuals involved, i.e., the dog owners, the clinicians involved in the intraoperative care (anaesthetist and surgeon), the postoperative investigator and the statistician, were blinded to the type of the intervention and unaware of patient allocation, to ensure that the results could not be influenced by the research team. A single surgeon performed all surgeries to ensure homogeneity regarding the surgical technique, and all postoperative pain assessments were conducted by the same experienced blinded evaluator to enhance scoring consistency and minimize interobserver variability. A validated pain scale was used, along with sedation assessment, to increase the sensitivity and specificity of postoperative pain detection and to ensure that the level of sedation could not influence pain assessment. Sedation and pain assessments were always performed in a standardized and consistent sequence, further ensuring controlled evaluation conditions. Randomization was applied also for group allocation, to minimize selection bias. Additionally, detailed and uniform intraoperative monitoring criteria were implemented to maintain comparable anaesthetic depth and ensure consistent detection and management of nociceptive responses. Magnesium concentrations were measured to quantify the extent of iatrogenic induced hypermagnesaemia, and potential magnesium-related muscle relaxation was assessed through postoperative patellar reflex evaluations. Other adverse effects potentially related to magnesium were systematically recorded to assess the feasibility and safety of the current magnesium dose regimen.

However, this study also has some limitations. First, the optimal analgesic dosing range of magnesium sulphate in dogs has not been established. The dosing regimen was extrapolated from human literature, and the limited veterinary studies available have not demonstrated clinically meaningful analgesic effects. Second, total serum magnesium concentrations were measured, rather than ionized magnesium, the biologically active fraction. Additional sampling points, such as immediately after the initial 50 mg kg^−1^ bolus and during the early post-infusion period (up to 4 hafter cessation), would have allowed more precise characterization of peak concentrations and elimination kinetics. The use of a preventive multimodal analgesic protocol including buprenorphine, meloxicam and paracetamol may also have limited the ability to detect any additional analgesic effect of magnesium, as the overall level of analgesia achieved may have reduced observable differences between groups in pain scores and rescue analgesia requirements. Although a standardized multimodal analgesic protocol was implemented, locoregional anaesthetic techniques were not included. This approach was intentionally selected to allow clearer evaluation of the adjunctive effect of IV magnesium, as the addition of local anaesthetic techniques could have reduced nociceptive input and potentially masked a modest treatment effect. However, their absence may limit direct extrapolation of the present findings to clinical settings where such techniques are routinely incorporated. Pre-induction haemodynamic variables were not recorded prior to administration of magnesium or saline. Therefore, potential baseline physiological differences between groups cannot be completely excluded, although randomization and protocol standardization were applied to minimize such variability. Furthermore, although spontaneous ventilation was intentionally permitted in accordance with the study objectives, standardized controlled ventilation for all dogs might have further reduced intraoperative variability in end-tidal CO_2_ and anaesthetic depth. The sample size represents an additional limitation. The power analysis was based exclusively on the CMPS-SF, and therefore secondary outcomes may be underpowered and should be interpreted cautiously. The absence of prior studies using pain as the primary endpoint for evaluating magnesium’s analgesic effects precluded the use of established effect-size estimates for more precise sample size calculation. This consideration is particularly relevant given the adjunctive nature of magnesium’s proposed analgesic effects. In addition, the analgesic effects of magnesium may be more pronounced in conditions associated with central sensitization or with the potential to transition to persistent postoperative pain, which were not represented in the present study. Finally, although postoperative pain and sedation were assessed by a single experienced blinded evaluator to ensure consistency across animals and timepoints, the absence of multiple independent assessors precluded formal assessment of interobserver agreement.

### 4.5 Clinical relevance and conclusions

Administration of magnesium sulphate (50 mg kg^−1^ loading dose followed by a 20 mg kg^−1^ h^−1^ infusion) during OVH in dogs, as part of a multimodal analgesic protocol, resulted in reduced pain scores at 1 h post-extubation; however, this analgesic effect was short-lived, and it did not reduce intraoperative or postoperative rescue analgesic requirements. Magnesium reduced propofol induction requirements but did not decrease isoflurane requirements for maintenance of anaesthesia. Although dogs in the Magnesium group exhibited slightly delayed extubation, no clinically significant cardiovascular, respiratory or other adverse effects were observed throughout the perioperative period, indicating that the current dose regimen is safe under the studied conditions. Overall, magnesium demonstrated modest perioperative effects and may serve as a potential adjunct within a multimodal analgesic strategy, rather than a primary analgesic intervention.

Further randomized studies are required to evaluate the analgesic efficacy of magnesium in surgical procedures involving different types of nociceptive stimuli, as well as in cases with pre-existing pain (indicating established NMDA receptor activation) or within opioid-free analgesia protocols. Future studies could also evaluate magnesium within protocols that incorporate locoregional anaesthetic techniques, to better reflect evolving clinical practice. Such investigations would allow a more accurate assessment of its effectiveness as an adjunct analgesic within a multimodal pain management framework in veterinary clinical practice. In addition, studies evaluating different dosing regimens are warranted to better define the optimal therapeutic range and clinical efficacy of magnesium in dogs.

## Data Availability

The raw data supporting the conclusions of this article will be made available by the authors, without undue reservation.
